# A family history of type 2 diabetes as a predictor of fatty liver disease in diabetes-free individuals with excessive body weight

**DOI:** 10.1038/s41598-021-03583-3

**Published:** 2021-12-16

**Authors:** Giovanni De Pergola, Fabio Castellana, Roberta Zupo, Sara De Nucci, Francesco Panza, Marco Castellana, Luisa Lampignano, Martina Di Chito, Vincenzo Triggiani, Rodolfo Sardone, Gianluigi Giannelli

**Affiliations:** 1 Unit of Geriatrics and Internal Medicine, National Institute of Gastroenterology “Saverio de Bellis”, Research Hospital, 70013 Castellana Grotte, BA Italy; 2 Unit of Data Sciences and Technology Innovation for Population Health, National Institute of Gastroenterology “Saverio de Bellis”, Research Hospital, 70013 Castellana Grotte, BA Italy; 3 Section of Internal Medicine, Geriatrics, Endocrinology, and Rare Disease, Interdisciplinary Department of Medicine, School of Medicine, University of Bari, 70124 Bari, Italy; 4 Scientific Direction, National Institute of Gastroenterology “Saverio de Bellis”, Research Hospital, 70013 Castellana Grotte, BA Italy

**Keywords:** Diseases, Endocrinology, Gastroenterology, Health care, Medical research, Risk factors

## Abstract

Comprehensive screening for non-alcoholic fatty liver disease (NAFLD) may help prompt clinical management of fatty liver disease. A family history, especially of diabetes, has been little studied as a predictor for NAFLD. We characterized the cross-sectional relationship between a family history of type 2 diabetes (FHT2D) and NAFLD probability in 1185 diabetes-free Apulian (Southern-Italy) subjects aged > 20 years with overweight or obesity not receiving any drug or supplementation. Clinical data and routine biochemistry were analysed. NAFLD probability was defined using the fatty liver index (FLI). A first-degree FHT2D was assessed by interviewing subjects and assigning a score of 0, 1, or 2 if none, only one, or both parents were affected by type 2 diabetes mellitus (T2DM). Our study population featured most females (70.9%, N = 840), and 48.4% (N = 574) of the sample had first-degree FHT2D. After dividing the sample by a FHT2D, we found a higher BMI, Waist Circumference (WC), and diastolic blood pressure shared by FHT2D subjects; they also showed altered key markers of glucose homeostasis, higher triglyceride levels, and worse liver function. FLI scores were significantly lower in subjects without a first-degree FHT2D. After running logistic regression models, a FHT2D was significantly associated with the NAFLD probability, even adjusting for major confounders and stratifying by age (under and over 40 years of age). A FHT2D led to an almost twofold higher probability of NAFLD, regardless of confounding factors (OR 2.17, 95% CI 1.63 to 2.89). A first-degree FHT2D acts as an independent determinant of NAFLD in excess weight phenotypes, regardless of the age group (younger or older than 40 years). A NAFLD risk assessment within multidimensional screening might be useful in excess weight subjects reporting FHT2D even in the absence of diabetes.

## Introduction

Research efforts are focused on charting clear directions in contexts of preventive medicine to anticipate major adverse health outcomes in adult populations. Along these lines, the global Burden of Disease epidemiological overview works well as a filter for areas of intervention. Thus, according to the latest register, multimorbidity and polypharmacy affect more than half of all adults, and chronic diseases top the list of health burdens to be faced^1^.

In this context, liver diseases have been recorded as one of the most alarming epidemiological data among non-communicable diseases in the last decade^1,2^. Non-alcoholic fatty liver disease (NAFLD) is currently among the most common metabolic liver disease, consisting of fat accumulation not attributable to secondary causes (e.g., significant alcohol consumption, viral infections, or medications). The latest reports indicate steadily increasing figures for NAFLD, affecting 20% of the general population and up to 70% of the population with diabetes^3,4^. However, the pathological course of NAFLD features a broad spectrum of features ranging from basic steatosis to steatohepatitis (NASH) or fibrosis, leading to cirrhosis and, in the worst cases, hepatocarcinoma. In this ruinous perspective, obese phenotypes with visceral ectopic fat accumulation, i.e., almost 80% of fatty liver phenotypes, carry a higher risk of liver damage^5^.

Beyond higher levels of liver enzymes, the pathophysiological pathways underlying NAFLD involve altered glucose homeostasis^6^; in this regard, we recently reported evidence of a possible bidirectional link whereby HbA1c acts as a collider^7^. As regards the direct association between exposure to glycaemic alterations and risk of NAFLD, a large body of evidence is provided in the literature, even in diabetes-free subjects^6,8^.

A further determinant of individual pathological risk trajectories is a family history of diabetes. A family history of type 2 diabetes (FHT2D) is a well-known strong risk factor for the onset of diabetes^9^ and is therefore included in many diabetes predictive models^10^. Of note, this phenomenon is dependent on the hereditary diabetic disease burden, so a FHT2D in both parents is more strongly associated with an impaired residual β-cell function^11–13^. Several studies in subjects with obesity demonstrated that a FHT2D accelerates the development of atherosclerosis among diabetes-free subjects. In particular, a FHT2D was found to increase the intima-media thickness of the common carotid artery, a surrogate marker of coronary atherosclerosis^14^ or the white blood cell count^15^, and to decrease sensitivity to activated protein C^16^. Moreover, there is consistent evidence that a FHT2D was associated with endothelial dysfunction^17^.

However, to date, little prospective investigation has explored the impact of a family history of diabetes on the risk of chronic disorders including liver disease. The multi-layered significance of the family clinical history does not aid a simple understanding of the underlying pathophysiological pathways, as diabetic familiarity depends on both heritable changes in gene expression and epigenetic driving forces. More than 75 independent genetic loci were found to be associated with type 2 diabetes mellitus (T2DM) in the first genome-wide association study (GWAS), and the epigenetic factor influenced by the surrounding environment also contributes significantly to the pathological endpoint^18^.

However, there is some evidence that a FHT2D is associated with higher fasting liver enzyme levels in the adult EPIC-Netherlands cohort without T2DM^19^; in this regard, it is not surprising that the authors found attenuation of this association after adjustment for diet, lifestyle, and adiposity, confirming the important role of these modifiable external factors. Furthermore, a multicenter cross-sectional study of adults with NAFLD diagnosed by abdominal ultrasound reported a FHT2D in 36.5% of participants^20^; more than 50% of these cases were complicated by overweight or obesity.

To our best knowledge, no study performed in a large southern Italian cohort in Europe has previously investigated whether a FHT2D is able to predict the NAFLD probability, regardless of the presence of diabetes and other confounding variables. The present research study, performed in 1185 Apulian (Southern-Italy) subjects with an excess weight phenotype but without diabetes, was conducted to examine any relationship between a first-degree FHT2D and NAFLD, independently of major glucose metabolism biomarkers potentially acting as confounding factors associated with NAFLD in excess weight phenotypes^7,21^. To note, the feature of native homogeneity of the population under study downplays selection bias in our survey, as it assumes a similar predisposition to environmental, lifestyle, and genetic risk factors.

## Methods

### Study population and design

**Figure 1:**
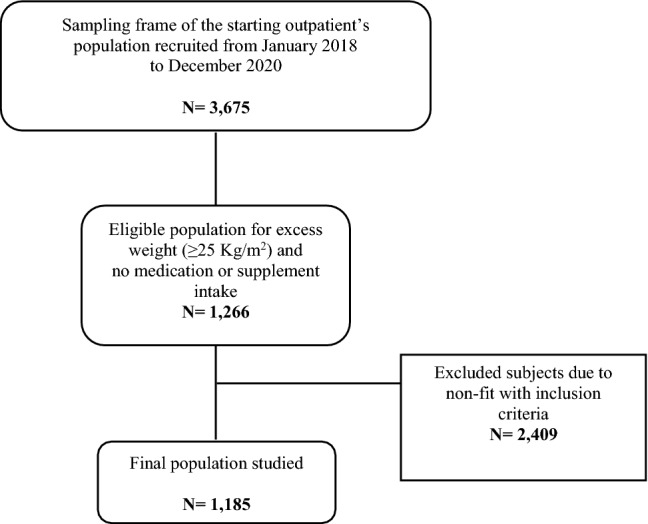
Flowchart of the population screening process.

From January 2018 to December 2020, in total, 3675 patients were recruited at the “Population Health Unit” of the National Institute of Gastroenterology “Saverio De Bellis”, Research Hospital (Castellana Grotte, Apulia, Italy). All data were collected at the baseline examination. Clinical variables were subsequently screened for adequacy against two inclusion criteria, i.e., BMI ≥ 25 kg/m^2^ and receiving no supplements or medication, including oral contraceptives or medicines for osteoporosis. Exclusion criteria were any endocrinological diseases (i.e., diabetes mellitus, hypo or hyperthyroidism, hypopituitarism), chronic inflammatory diseases, stable hypertension, angina pectoris, a history of stroke, transient ischaemic attack, atrial fibrillation, heart infarction, congenital heart disease, any major malignancies, kidney or liver failure, inherited thrombocytopenia, HBV or HCV infections, and excess dietary alcohol intake. After excluding subjects not satisfying the inclusion criteria, the final study population consisted of 1185 subjects (889 females, 377 males) aged > 20 years. A summary flowchart of the population screening process is shown in Fig. 1. The study protocol (ClinicalTrials.gov Identifier: NCT04327375) met the principles in the Declaration of Helsinki and was approved by the Ethics Committee of the National Institute of Gastroenterology “S. De Bellis” Research Hospital (Castellana Grotte, Apulia, Italy). All participants gave informed consent before enrolment. Analyses were conducted in January 2021.

### Clinical examination and fluid biomarkers collection

At baseline, metabolic and routine biochemistry parameters were closely examined in all subjects. A brief interview, including questions about medical history and lifestyle, was conducted by a senior physician. Information about diabetic familiarity was collected by interviewing subjects and assigning a score of 0, 1, or 2 if none, only one, or both parents were affected by type 2 diabetes mellitus (T2DM). Information on daily alcohol consumption was assessed by direct questioning while taking the medical history, as follows: “do you drink at least two glasses of alcohol per day?” for males or “do you drink at least one glass of alcohol per day?” for females. In accordance with the American and European guidelines for daily alcohol consumption^22,23^, suggesting a threshold of 20 g/day in females and 30 g/day in males, those subjects reporting a positive response were excluded from the final study population. Extemporaneous outpatients’ diastolic (DBP) and systolic blood pressure (SBP) was determined in a sitting position after at least a 10-min rest, a minimum of three different times, using an OMRON M6 automatic Blood Pressure monitor. A smoking habit was also investigated as a dichotomous variable (yes/no). Blood samples were drawn at 08.00–09.00 am, after overnight fasting. The blood cell count was determined with a Coulter Hematology analyzer (Beckman–Coulter, Brea, CA). Fasting plasma glucose (FPG), HbA1c, insulin, total cholesterol, high-density lipoprotein (HDL) cholesterol, triglycerides, and liver markers serum levels were assayed. Serum insulin concentrations were measured by radioimmunoassay (Behring, Scoppito, Italy), and all samples were analyzed in duplicate. Fasting plasma glucose was determined using the glucose oxidase method (Sclavus, Siena, Italy), while the concentrations of plasma lipids (triglycerides, total cholesterol, HDL cholesterol) were quantified by an automated colorimetric method (Hitachi; Boehringer Mannheim, Mannheim, Germany). HbA1c was routinely assayed on a chemical analyzer Architect c8000 (Abbott). Amino transferase and gamma-glutamyl transpeptidase (γGT) were measured with standard routine laboratory methods. Low-density lipoprotein (LDL) cholesterol was calculated using the Friedewald equation^24^. Insulin resistance was assessed using the homeostasis model assessment–insulin resistance (HOMA-IR)^25^.

### Anthropometric assessment

Two qualified nutritionists (RZ, LL), trained for equivalent measuring performances, carried out clinical procedures. All anthropometric measurements were taken with participants dressed in lightweight clothing and without shoes. Variables were all collected simultaneously at 7.00–10.00 am, after overnight fasting. Height was measured to the nearest 0.5 cm using a wall-mounted stadiometer (Seca 711; Seca, Hamburg, Germany). Bodyweight was determined to the nearest 0.1 kg using a calibrated balance beam scale (Seca 711; Seca, Hamburg, Germany). BMI was calculated by dividing body weight (Kg) by the square of height (m^2^) and classified according to World Health Organization criteria for normal weight (18.5–24.9 kg/m^2^), overweight (25.0–29.9 kg/m^2^), grade I obesity (30.0–34.9 kg/m^2^), grade II obesity (35.0–39.9 kg/m^2^), and grade III obesity (≥ 40.0 kg/m^2^)^26^. Waist circumference (WC) was measured at the narrowest part of the abdomen or in the area between the tenth rib and the iliac crest (minimum circumference).

### NAFLD risk assessment

The FLI, a modelling algorithm including BMI, WC, triglycerides, and γGT^27^, was used to assess the NAFLD probability. The calculation was made according to the following equation: (e 0.953 × loge (TG) + 0.139 × BMI + 0.718 × loge (GGT) + 0.053 × WC—15.745)/(1 + e 0.953 × loge (TG) + 0.139 × BMI + 0.718 × loge (GGT) + 0.053 × WC—15.745) × 100. Subjects with FLI < 30 are classified as at low risk of NAFLD, and those with FLI ≥ 60 at high risk.

### Statistics

We performed descriptive analysis of baseline variables, expressed as mean ± Standard Deviation (SD), and proportion (%) for the frequency of categorical variables. First the sample was divided into two categories, according to a positive or negative FHT2D, and subsequently we performed the same descriptive analysis on three categories, to appreciate the differences between subjects with only one diabetic parent or both. The normality of distribution was assessed for each variable using Shapiro’s test. A non-parametric approach was used to assess statistically significant differences between groups for all collected variables. The distribution of each variable was reported as mean ± SD for continuous variables, and as proportion (%) to facilitate comparison between our study and others, as these are more generalizable and reliable measures than median and range. Mann Whitney sum rank test was used to assess statistically significant differences between groups for continuous variables, and Chi square test for categorical variables. p-values less than or equal to 0.05 were considered statistically significant, with 95% confidence intervals. A Spearman’s correlation matrix was built for all continuous biochemical and anthropometric variables to check for interrelated variables and so avoid collinearity effects in the model (Supplementary Table S1). Logistic regression models were performed on a single or dual first-degree FHT2D and the probability of NAFLD, also subdividing the sample by age groups, i.e., under and over 40 years of age. Several nested hierarchical models were built to assess the association independently of confounding factors: (1) raw model using only FHT2D as the main covariate, (2) model 1 plus age, sex, and smoking, (3) model 2 plus FBG, (4) model 3 plus insulin levels. The methodological approach and analyses were designed and managed by a senior epidemiologist (RS) and a biostatistician (FC) using RStudio software, version 1.4.1106^28^.

## Results

The examined population (N = 1185) was dominated by females (N = 840, 70.90%); mean age was 47 years, ranging from 20 to 74. A comparative descriptive analysis of the sample by a first-degree FHT2D (yes if subjects had at least one parent with T2DM, otherwise no) is shown in Table 1. In total, 574 (48.4%) subjects in the population had at least one parent with diabetes, while only 65 (5.5%) had both. A greater BMI (*p* < 0.01), WC (*p* < 0.01), and extemporaneous diastolic BP (*p* = 0.03) were found to be clinical features common to subjects with at least one diabetic parent. These subjects also exhibited poorer levels of major glycaemic metabolism parameters, i.e., FBG, insulin, HOMA index, and HbA1c (p < 0.01). As to lipid metabolism, the same subjects presented significantly higher levels of triglycerides (p < 0.01), but no significant differences compared to the FHT2D group for total- (p = 0.49), HDL- (p = 0.26), and LDL-cholesterol (p = 0.92) levels. When considering liver function parameters, however, the FHT2D group had significantly higher mean levels of circulating AST (p = 0.04), ALT (p < 0.01), and gamma-GT (p < 0.01). Thus, the FLI score was significantly higher in subjects who had at least one parent affected by T2DM (p < 0.01), and a pathological value (FLI ≥ 60) was found to be highly prevalent in the same subjects group (N = 370, 60.6%) as compared with those without a FHT2D (p < 0.01). Table 2 shows the same comparative analyses of investigated parameters after dividing the whole population into three categories, based on whether none, only one, or both parents had T2DM. Differences in mean values demonstrate significance for those same parameters pointed out in the first analysis. Dual familiarity, i.e., both parents with diabetes, did not seem to increase the NAFLD probability.

**Table 1:** Description of the whole sample according to FHT2D.

Proportion (%)	Negative family history		Positive family history		p value*
	611 (51.60)		574 (48.40)		
	Mean ± SD	Median (min to max)	Mean ± SD	Median (min to max)	
Age (years)	39.84 ± 12.38	40 (20 to 72)	41.00 ± 11.34	41 (20 to 74)	0.07
**Age groups**					
Under 40 years	304 (49.80)		271 (47.20)		0.38 χ^2^
Over 40 years	307 (50.20)		303 (52.80)		
**Sex**					
Female	437 (71.50)		403 (70.20)		0.61 χ^2^
Male	174 (28.50)		171 (29.80)		
Smoking (yes)	129 (21.80)		114 (20.70)		0.64 χ^2^
BMI (kg/m^2^)	32.9 ± 5.94	31.5 (22.6 to 65)	34.94 ± 6.31	33.81 (25.2 to 79)	** < 0.01**
WC (cm)	106.13 ± 14.1	104 (69 to 158)	111.14 ± 14.1	110 (82.5 to 193)	** < 0.01**
SBP (mmHg)	125.72 ± 13.99	125 (86 to 170)	125.82 ± 14.05	125 (90 to 170)	0.75
DBP (mmHg)	80.98 ± 9.94	80 (55 to 120)	81.84 ± 9.01	80 (57 to 110)	0.10
FBG (mg/dl)	89.97 ± 9.91	89 (65 to 125)	92.69 ± 10.94	92 (68 to 125)	** < 0.01**
Insulin (UI)	20.82 ± 15.39	17 (2.4 to 128)	25.04 ± 16.68	20.8 (3.7 to 119)	** < 0.01**
HOMA-IR	4.72 ± 3.79	3.89 (0.52 to 30.14)	5.82 ± 4.08	4.58 (0.72 to 27)	** < 0.01**
HbA1c (%)	5.32 ± 0.33	5.3 (4.35 to 6.45)	5.41 ± 0.41	5.38 (4.6 to 6.3)	0.09
Triglycerides (mg/dl)	103.79 ± 58.27	92 (23 to 408)	112.07 ± 64.54	97 (23 to 541)	**0.01**
HDL cholesterol (mg/dl)	49.01 ± 12.74	48 (21 to 95)	48.03 ± 12.48	47 (19 to 111)	0.22
Total cholesterol (mg/dl)	193.18 ± 38.68	191 (51 to 330)	194.19 ± 38.42	193 (97 to 372)	0.71
LDL cholesterol (mg/dl)	123.93 ± 33.03	121 (37 to 240)	124.27 ± 34.09	121 (23 to 262)	0.93
Platelets (10^3^ cells/mm^3^)	265.06 ± 62.65	261 (2.94 to 517)	261.59 ± 60.89	257.5 (101 to 568)	0.18
AST (U/l)	22.16 ± 8.43	20 (8 to 71)	23.32 ± 9.34	21 (9 to 85)	**0.01**
ALT (U/l)	41.3 ± 20.96	38 (9 to 201)	45.31 ± 21.71	41 (10 to 172)	** < 0.01**
Gamma GT (U/l)	31.67 ± 23.42	25 (9 to 238)	35.86 ± 26.46	28 (5 to 290)	** < 0.01**
FLI (%)	65.22 ± 27.53	70.5 (3.59 to 99.98)	76.62 ± 21.41	81.72 (11.15 to 100)	** < 0.01**
FLI ≥ 60 (%)	370 (60.60)		460 (80.10)		** < 0.01** χ^2^

N: 1185.

All data are shown as mean ± SD for continuous variable and as n (%) for proportions.

*FHT2D* family history of type 2 diabetes, *BMI* body mass index, *WC* waist circumference, *SBP* systolic blood pressure, *DBP* diastolic blood pressure, *FBG* fasting blood glucose, *HOMA-IR* homeostasis model assessment–insulin resistance, *AST* aspartate amino transferase, *ALT* alanine amino transferase, *γGT* gamma-glutamyl transpeptidase, *FLI* fatty liver index.

*Mann Whitney sum rank test, χ^2^ Chi squared test.

Significant values are given in bold.

**Table 2:** Description of the whole sample according to FHT2D.

Proportion (%)	Negative family history		Positive family history (one parent)		Dual family history (both parents)		p value
	592 (51.80)		489 (42.80)		62 (5.40)		
	Mean ± SD	Median (min to max)	Mean ± SD	Median (min to max)	Mean ± SD	Median (min to max)	
Age (years)	39.84 ± 12.38	40 (20 to 72)	40.89 ± 11.38	41 (20 to 69)	41.86 ± 11.05	40 (20 to 74)	0.18
**Sex**							
Female	437 (71.50)		354 (69.50)		49 (75.40)		0.54 χ^2^
Male	174 (28.50)		155 (30.50)		16 (24.60)		
Smoking (yes)	129 (21.80)		103 (21.10)		11 (17.70)		0.75 χ^2^
BMI (kg/m^2^)	32.9 ± 5.94	31.5 (22.6 to 65)	35.05 ± 6.46	33.9 (25.2 to 79)	34.05 ± 4.91	33.3 (26.9 to 49.71)	** < 0.01**
WC (cm)	106.13 ± 14.1	104 (69 to 158)	111.38 ± 14.44	110 (82.5 to 193)	109.28 ± 11.06	109 (86 to 144)	** < 0.01**
SBP (mmHg)	125.72 ± 13.99	125 (86 to 170)	125.52 ± 14.12	125 (90 to 170)	128.2 ± 13.3	130 (99 to 167)	0.25
DBP (mmHg)	80.98 ± 9.94	80 (55 to 120)	81.66 ± 9.1	80 (57 to 110)	83.23 ± 8.25	80 (65 to 105)	0.12
FBG (mg/dl)	89.97 ± 9.91	89 (65 to 125)	92.42 ± 11	91 (68 to 125)	94.72 ± 10.3	95 (79 to 124)	** < 0.01**
Insulin (UI)	20.82 ± 15.39	17 (2.4 to 128)	25.24 ± 16.84	20.6 (3.7 to 119)	23.48 ± 15.45	21 (4.6 to 70)	** < 0.01**
HOMA-IR	4.72 ± 3.79	3.89 (0.52 to 30.14)	5.85 ± 4.1	4.59 (0.72 to 27)	5.56 ± 3.97	4.46 (0.91 to 21.4)	** < 0.01**
HbA1c (%)	5.32 ± 0.33	5.3 (4.35 to 6.45)	5.39 ± 0.4	5.35 (4.6 to 6.3)	5.59 ± 0.42	5.7 (4.6 to 6.2)	**0.02**
Triglycerides (mg/dl)	103.79 ± 58.27	92 (23 to 408)	113.06 ± 64.24	98 (24 to 541)	104.29 ± 66.85	90 (23 to 400)	**0.01**
HDL cholesterol (mg/dl)	49.01 ± 12.74	48 (21 to 95)	47.92 ± 12.4	46 (19 to 102)	48.95 ± 13.14	48 (25 to 111)	0.39
Total cholesterol (mg/dl)	193.18 ± 38.68	191 (51 to 330)	194.16 ± 38.79	192 (97 to 372)	194.43 ± 35.72	194 (118 to 298)	0.91
LDL cholesterol (mg/dl)	123.93 ± 33.03	121 (37 to 240)	123.97 ± 33.98	119 (32 to 233)	126.61 ± 35.09	128 (23 to 262)	0.70
Platelets (10^3^ cells/mm^3^)	265.06 ± 62.65	261 (2.94 to 517)	261.42 ± 61.14	257 (101 to 568)	262.95 ± 59.35	258 (149 to 464)	0.41
AST (U/l)	22.16 ± 8.43	20 (8 to 71)	23.47 ± 9.15	22 (9 to 81)	22.15 ± 10.75	20 (13 to 85)	** < 0.01**
ALT (U/l)	41.3 ± 20.96	38 (9 to 201)	45.85 ± 21.52	41 (10 to 172)	41.06 ± 22.82	33 (15 to 151)	** < 0.01**
Gamma GT (U/l)	31.67 ± 23.42	25 (9 to 238)	36.14 ± 27.05	28 (5 to 290)	33.65 ± 21.32	28 (11 to 115)	** < 0.01**
FLI (%)	65.22 ± 27.53	70.5 (3.59 to 99.98)	76.9 ± 21.51	82.17 (11.15 to 100)	74.42 ± 20.65	79.37 (14.52 to 99.9)	** < 0.01**
FLI ≥ 60 (%)	370 (60.60)		410 (80.60)		50 (76.90)		** < 0.01** χ^2^

N = 1185.

All data are shown as mean ± SD for continuous variable and as n (%) for proportions.

*FHT2D* family history of type 2 diabetes, *BMI* body mass index, *WC* waist circumference, *SBP* systolic blood pressure, *DBP* diastolic blood pressure, *FBG* fasting blood glucose, *HOMA-IR* homeostasis model assessment–insulin resistance, *AST* aspartate amino transferase, *ALT* alanine amino transferase, *γGT* gamma-glutamyl transpeptidase, *FLI* fatty liver index.

*Kruskal Wallis sum rank test and χ^2^ Chi squared test.

Significant values are given in bold.

Table 3 shows results of logistic regression models for the NAFLD probability (FLI ≥ 60), hierarchically adjusted for selected confounding factors, i.e., (1) raw model using only FHT2D as the main covariate, (2) model 1 plus age, sex, and smoking, (3) model 2 plus FBG, (4) model 3 plus insulin levels. A FHT2D proved to be independently associated with the NAFLD probability, even after adjusting major glucose metabolism biomarkers. In practice, a first-degree FHT2D was linked to a twofold higher probability of carrying an increased risk of liver steatosis, regardless of confounding factors (OR 2.17, 95% CI 1.63 to 2.89). Table 4 shows the results of full adjusted logistic regression models subdivided by age groups, i.e., 20–40 years and 40–74 years of age. Again, a FHT2D proved to be independently associated with the NAFLD probability in both groups (OR 3.45, 95% CI 2.22 to 5.35 and OR 1.48, 95% CI 1.1 to 2.19, respectively among under and over 40 years of age groups). The effect is much greater in those under 40 probably due to the smaller number of subjects, highlighted by a larger CI.

**Table 3:** Logistic regression models on FLI ≥ 60% as dependent variable.

	OR	CI 95%	p value
**Model 1**			
FHT2D	2.63	2.02 to 3.41	** < 0.01**
**Model 2**			
FHT2D	2.64	2.02 to 3.45	** < 0.01**
Age (over 40 years)	1.01	0.78 to 1.31	0.95
Sex (female)	1.01	0.75 to 1.34	0.97
Smoking (yes)	0.90	0.66 to 1.23	0.51
**Model 3**			
FHT2D	2.43	1.84 to 3.2	** < 0.01**
Age (over 40 years)	0.73	0.56 to 0.97	**0.02**
Sex (female)	1.02	0.76 to 1.37	0.91
Smoking (yes)	0.84	0.61 to 1.16	0.29
FBG (mg/dl)	1.06	1.05 to 1.08	** < 0.01**
**Model 4**			
FHT2D	2.17	1.63 to 2.89	** < 0.01**
Age (over 40 years)	1.01	0.74 to 1.34	0.98
Sex (Female)	0.99	0.73 to 1.35	0.95
Smoking (yes)	0.85	0.6 to 1.19	0.34
FBG (mg/dl)	1.05	1.03 to 1.06	** < 0.01**
Insulin (UI)	1.06	1.05 to 1.08	** < 0.01**

Model 1: Raw Model.

Model 2: corrected for age, sex and smoking.

Model 3: corrected for age, sex, smoking and Fasting Blood Glucose levels.

Model 4: corrected for age, sex, smoking, Fasting Blood Glucose and insulin levels).

*FHT2D* family history of type 2 diabetes, *FLI* fatty liver index, *FBG* fasting blood glucose.

Significant values are given in bold.

**Table 4:** Full adjusted logistic regression models on FLI ≥ 60% as dependent variable subdivided by age groups.

	OR	CI 95%	p value
**Under 40 years**			
FHT2D	3.45	2.22 to 5.35	** < 0.01**
Sex (female)	1.39	0.89 to 2.16	0.15
Smoking (yes)	0.84	0.51 to 1.38	0.49
FBG (mg/dl)	1.05	1.02 to 1.07	** < 0.01**
Insulin (UI)	1.06	1.04 to 1.08	** < 0.01**
**Over 40 years**			
FHT2D	1.48	1.1 to 2.19	**0.04**
Sex (female)	0.73	0.47 to 1.13	0.15
Smoking (yes)	0.86	0.54 to 1.39	0.54
FBG (mg/dl)	1.05	1.03 to 1.07	** < 0.01**
Insulin (UI)	1.07	1.04 to 1.09	** < 0.01**

*FHT2D* family history of type 2 diabetes, *FBG* fasting blood glucose.

Significant value are given in bold.

## Discussion

The present study examined a large population of diabetes-free subjects with overweight or obesity, providing evidence of a close positive cross-sectional relationship between a FHT2D and the NAFLD probability, regardless of age group (younger or older than 40 years) (Table 4). These findings stand as seminal and lay the foundation for further research that could focus on longitudinal observation of the mechanisms of glucose metabolism as well as fluctuations in liver enzymes in relation to the presence of FHT2D.

A body of evidence has already shown how in subjects with NAFLD and a pattern of blood chemistry consisting of both elevated hepatic and glycaemic biomarkers can be predictive of diabetes, and how high HbA1c levels are associated with NAFLD in non-diabetic phenotypes^6,8^. Our study fits into this context by further adding one novel aspect, as we illustrate that NAFLD risk may also be explained by FHT2D in the absence of diabetes and any pharmacological therapy, based on a large cohort analysed here. Furthermore, the analysis was performed within a homogeneous cohort from southern Italy in Europe that shares similar characteristics.

This finding, obtained using the FLI, a scoring algorithm based on BMI, WC, and serum γGT and triglycerides levels, appears to be in line with a previous report showing that a family history of T2DM was associated with higher fasting liver enzyme levels in a general diabetes-free population^19^. This information may be handy in the clinical setting; indeed, while a family history of T2DM was already acknowledged to be a major risk factor for the onset of diabetes^9,10^ as well as in speeding up atherosclerotic events in diabetes-free subjects^12–15^, we now add the unfavourable new finding of an increased probability of developing NAFLD. Practically speaking, it will be helpful to stress the importance for healthcare clinicians of possibly including a thorough NAFLD risk assessment within a multidimensional screening program for excess weight subjects reporting a FHT2D.

Consistent with this preliminary result, we found impaired glucose metabolism biomarkers, i.e., FBG, insulin, HOMA-IR, and HbA1c, to be clinical features common to FHT2D subjects. Aligned with the presence of insulin resistance, significantly higher triglycerides values were found in the same group. Most interestingly, the association between a FHT2D and the NAFLD probability in this study was corroborated independently of age, sex, and all factors well-known to be related to NAFLD (Table 3) such as smoking, FBG, and insulin^7^, thus reinforcing the importance of investigating a FHT2D when caring for individuals with overweight or obesity and free from any drug therapy. Indeed, among these major confounders considered, there is evidence that genetic polymorphisms, sex hormones, and dysmetabolic traits work synergistically well in delineating a female prevalence of NAFLD^29^. Smoking was also considered in corroborating our findings, consistent with evidence defining smoking habit among major risk factors associated with NAFLD and the most recent metrics indicating approximately 30–40% prevalence of NAFLD among smokers^30^. It should be noted as well that the association was maintained even after adjustment for age, though the prevalence of NAFLD is known to be negatively affected by aging^31^; this aspect further strengthens the main association, stressing FHT2D as a matter of consideration.

Notably, further analysis of our study population showed that a dual FHT2D, i.e., both parents with T2DM, did not further increase the likelihood of NAFLD, suggesting that, in some way, lack of a FHT2D might offer some safety against the development of steatosis by protecting against insulin resistance and impaired glucose metabolism.

Pending further investigation to explain the possible mechanisms underlying our main finding, preventive intervention to tackle the risk of NAFLD is key when examining FHT2D subjects. By targeting familiarity, and being aware that both genetic and environmental factors can weigh on it, acting on the lifestyle front is clearly a good idea. Among preventive strategies, recent longitudinal data are encouraging regarding the efficacy of lifestyle interventions. Specifically, Zhu and colleagues demonstrated that patients with a family history of T2DM and metabolic syndrome benefited more significantly from lifestyle interventions in terms of insulin resistance than those without a FHT2D, regardless of body weight changes, over a 2-year follow-up^8,32^.

### Limitations

Some study limitations must be considered. Because of the cross-sectional design, we cannot appreciate the temporal nature of the associations, so prospective studies are needed to clarify any causal relationship. Moreover, NAFLD was estimated using a probability scoring tool rather than validated imaging techniques for the diagnosis of NAFLD, although this scoring is still the only guideline-recommended tool for evaluating liver steatosis when imaging and biopsy are not available^29^. An important limitation is the lack of information on parental lifestyle, assuming that the pattern of association is not only a genetic but also an epigenetic mechanism. Therefore, it would have been useful to investigate parental behavioral and social factors, as well as diet habits. The main strength of this study is that it included only individuals not taking medications, thus avoiding any possible interference with biomarkers testing and survey results.

## Conclusions

We conclude that a first-degree FHT2D acts as an independent determinant of the NAFLD probability in excess weight phenotypes, markedly worsening the adverse trajectories of overall health status that are commonly attributed to impacts on glucose metabolism. As a consequence, the inclusion of an accurate NAFLD risk assessment within multidimensional screening for overweight individuals reporting a FHT2D becomes a crucial point whose importance needs to be highlighted.

## Supplementary Information

Supplementary Table S1.

## Data Availability

Data are available on request from the authors.
