# Supplementary material for: A family history of type 2 diabetes as a predictor of fatty liver disease in diabetes-free individuals with excessive body weight
Source: Sci Rep. 2021 Dec 16;11:24084. doi: 10.1038/s41598-021-03583-3 (PMC8677812; doi:10.1038/s41598-021-03583-3)
Supplement: Supplementary file 1 — Supplementary Table S1. [file 41598_2021_3583_MOESM1_ESM.docx]

**Supplementary Table 1**. Spearman’s correlation matrix of Fatty Liver Index (FLI) and other continuous collected variables.

|  | **Rho** | **p value** |
| --- | --- | --- |
| Age (years) | 0.01 | 0.75 |
| BMI (Kg/m^2^) | 0.84 | **<0.01** |
| WC (cm) | 0.90 | **<0.01** |
| SBP (mmHg) | 0.24 | **<0.01** |
| DBP (mmHg) | 0.21 | **<0.01** |
| FBG (mg/dl) | 0.29 | **<0.01** |
| Insulin (UI) | 0.47 | **<0.01** |
| HOMA-IR | 0.49 | **<0.01** |
| HbA1c (%) | 0.14 | **0.02** |
| Triglycerides (mg/dl) | 0.57 | **<0.01** |
| HDL Cholesterol (mg/dl) | -0.39 | **<0.01** |
| Total Cholesterol (mg/dl) | 0.14 | **<0.01** |
| LDL Cholesterol (mg/dl) | 0.14 | **<0.01** |
| Platelets (10^3^ cells/mm^3^) | 0.07 | **0.02** |
| AST (U/L) | 0.36 | **<0.01** |
| ALT (U/L) | 0.41 | **<0.01** |
| Gamma GT (U/L) | 0.55 | **<0.01** |
| FLI (%) | 1 | NA |

***Abbreviations****: BMI (Body Mass Index); WC (Waist Circumference); SBP (Systolic Blood Pressure); DBP (Diastolic Blood Pressure); FBG (Fasting Blood Glucose); HOMA-IR (Homeostasis Model Assessment – Insulin Resistance); AST (Aspartate Amino Transferase); ALT (Alanine Amino Transferase); γGT (gamma-Glutamyl Transpeptidase); FLI (Fatty Liver Index).*
